# Health Literacy: A Policy Imperative for Enhancing Heart Failure Care

**DOI:** 10.34172/hpp.45385

**Published:** 2026-08-21

**Authors:** Arash Ziapour

**Affiliations:** ^1^Cardiovascular Research Center, Health Policy and Promotion Institute, Kermanshah University of Medical Sciences, Kermanshah, Iran; ^2^Research Center for Environmental Determinants of Health (RCEDH), Health Institute, Kermanshah University of Medical Sciences, Kermanshah, Iran

**Keywords:** Health literacy, Heart failure, Health policy, Medication adherence, Patient education

## Abstract

Health literacy is a critical determinant of effective heart failure (HF) management and an essential policy priority for improving patient outcomes. Limited health literacy impairs disease understanding, self-care, treatment adherence, and quality of life while increasing healthcare utilization and hospital readmissions. This policy brief is informed by findings from a cross-sectional study of 385 patients with heart failure in northwest Iran. Health literacy was assessed using the Functional, Communicative, and Critical Health Literacy (FCCHL) scale. Descriptive statistics and Pearson correlation analyses were performed to examine health literacy levels and their associations with key patient outcomes. The findings identified substantial deficiencies in health literacy among patients with heart failure, highlighting significant barriers to effective disease management and implementation of evidence-based cardiac care policies. Lower health literacy was associated with poorer self-care, reduced treatment adherence, and less favorable health outcomes, emphasizing the need for health literacy–responsive healthcare systems.

**Implications for Policy:**

Strengthening health literacy should be a national policy priority in heart failure care. Integrating structured health literacy interventions into routine clinical practice, implementing targeted patient education, and addressing social determinants of health can improve self-care, medication adherence, health equity, and quality of life. Evidence from the Heart and Stroke Foundation of Canada indicates that post-discharge health literacy education clinics reduced hospital readmissions by 22%. Policy implementation should be accompanied by measurable indicators, including a 15% reduction in readmission rates within 12 months, improved medication adherence assessed using the Medication Adherence Report Scale (MARS), and periodic evaluation of patient health literacy using the FCCHL instrument. Immediate integration of health literacy into cardiac care frameworks is essential to reduce disparities and improve cardiovascular outcomes.

## The Policy Problem

 Heart failure affects more than 64 million individuals worldwide^1^ and represents one of the major global public health challenges with significant clinical and economic consequences. Shifting the care paradigm for patients with heart failure from a “treatment-oriented” approach to an “empowerment model based on health literacy” can play a decisive role in improving quality of life, reducing hospital readmissions, and enhancing patients’ adherence to treatment. ^2,3^ Heart failure poses a significant public health challenge in Iran, marked by its rising prevalence and substantial economic impact. Approximately 3.3% of the adult population in Iran is affected by this condition.^4^ Recent data from various studies indicate a range of age-standardized prevalence rates of cardiovascular diseases (CVDs) per 100,000 individuals. A global age-standardized prevalence rate of 7,179 cases per 100,000 individuals was reported in 2021.^5^ In Iran, the age-standardized prevalence rate remained stable nationally, ranging from 9,956 to 10,386 per 100,000 in 2021, with males showing higher rates than females.^6^ Despite advancements in medical treatment for this condition, significant gaps remain in Iran’s health policies, especially regarding health literacy, which impede the effective management of heart failure.^7^

 Research indicates that a significant proportion of cardiac patients in Iran, particularly those with heart failure and ischemic heart disease, exhibit inadequate or moderate health literacy. This is consistently associated with poorer self-care, lower medication adherence, increased hospital readmissions, and elevated mortality risk.^4,8^ In contrast to several developed countries that have implemented structured health literacy education programs within their healthcare systems, Iran currently lacks a comprehensive national framework for assessing and promoting health literacy among patients with chronic diseases, such as heart failure. These policy gaps set the stage for targeted interventions (Table 1).

**Table 1:** Summary of Key Gaps in Iran’s Health Literacy Policies for Heart Failure Management

**Key Gap**	**Explanation **	**Consequence**
Absence of a comprehensive national framework	Absence of a health literacy assessment and promotion program for chronic diseases, including heart failure.	Increased readmission rates of up to 30% and rising treatment costs.
Insufficient health literacy	Seventy-four percent of heart patients demonstrate inadequate health literacy, suggesting that focusing solely on individual factors, without complementary policy interventions, may be insufficient.	Reduced medication adherence and an elevated risk of preventable hospitalizations.
Inequities in access and coverage	Insufficient healthcare services in rural areas and inadequate insurance coverage for health literacy education.	Increased prevalence of preventable complications and enduring social inequalities.
Deficiency of evidence-based interventions	Insufficient sustainability of interventions and inadequate evaluation, along with an emphasis on health technology assessment (HTA) that lacks the integration of health literacy.	Challenges in self-management and deficiencies in non-communicable disease (NCD) policies

According to the World Health Organization, health literacy is a critical social determinant of health that significantly influences individuals’ capacity to make informed decisions and engage in self-management behaviors.^9^ This definition positions health literacy as an organizational mediator in the management of chronic diseases, including heart failure.^10^

 According to the World Health Organization, health literacy is a critical social determinant of health that significantly influences individuals’ capacity to make informed decisions and engage in self-management behaviors.^9^ This definition positions health literacy as an organizational mediator in the management of chronic diseases, including heart failure (Figure 1).^10^

**Figure 1:**
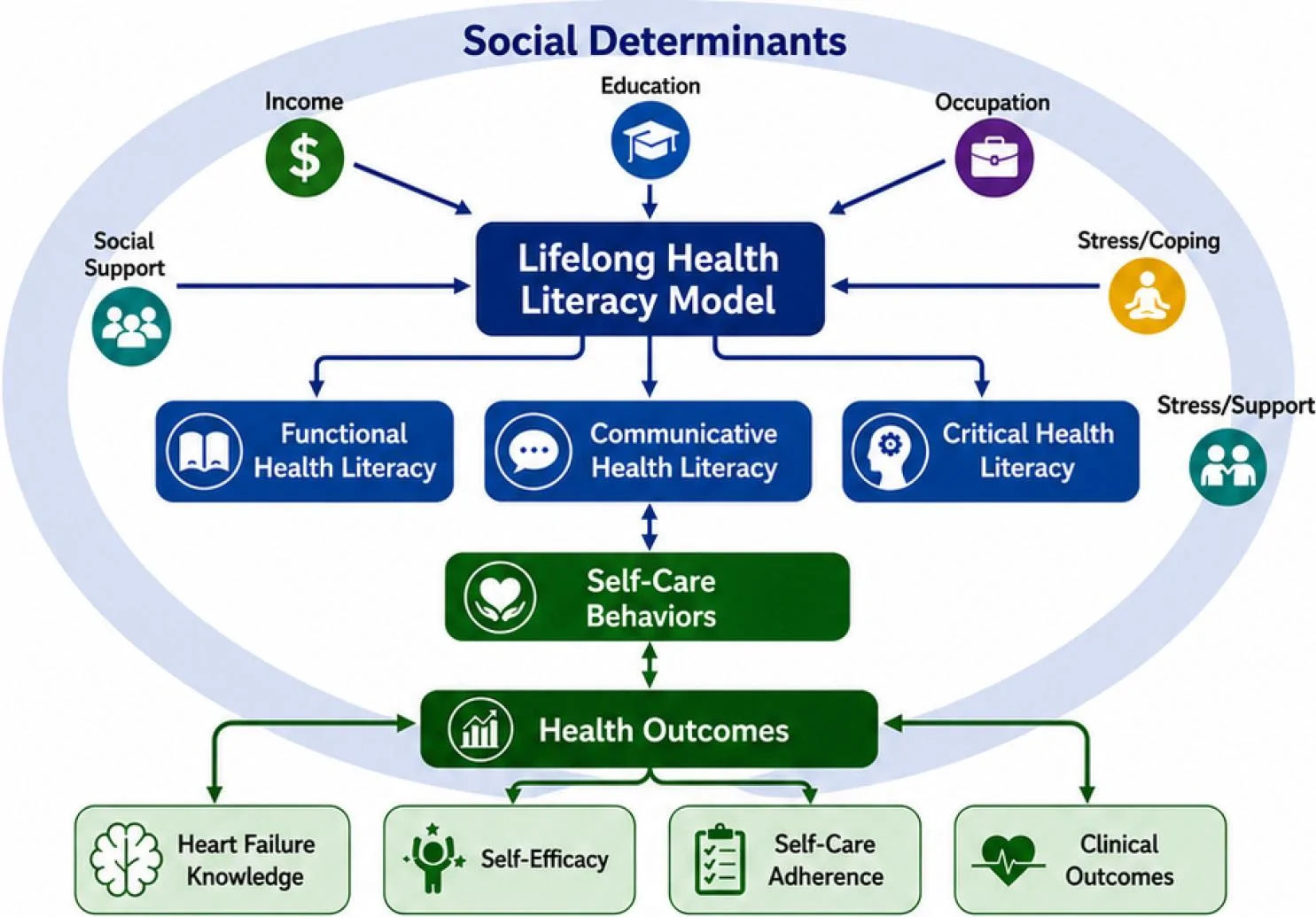
Cyclic pathway of health literacy to heart failure outcomes: Integrating social factors, a lifelong model, and triple skills

 Health literacy, defined as the ability to access, understand, appraise, and apply health information, is critical for effective self-management and clinical outcomes in patients with heart failure. ^11^ Research indicates that patients with higher health literacy levels experience fewer concerns related to heart failure management, possess a better understanding of healthcare access, and report improved quality of life.^12^ Given the significant prevalence of low health literacy among patients with HF, it is essential for nurses and healthcare professionals to recognize its detrimental effects and implement strategies to mitigate these impacts on patient health outcomes.^13,14^ These results are consistent with the European Union’s policy framework, which emphasizes the integration of health literacy into chronic disease management systems. This framework aims to enhance health literacy, improve health outcomes, and reduce health disparities associated with noncommunicable diseases across Europe.^15^ This initiative facilitates the integration of health literacy into the management of noncommunicable diseases, including heart failure.^15^ Enhancing health literacy can facilitate improved decision-making regarding disease treatment and elevate the quality for life for patients with chronic illnesses, including heart failure.^16^ Furthermore, the advancement of health literacy is regarded as fundamental to the prevention and control of noncommunicable diseases and should serve as a foundation for local and national policies designed to develop supportive environments (Figure 2).^17^

**Figure 2:**
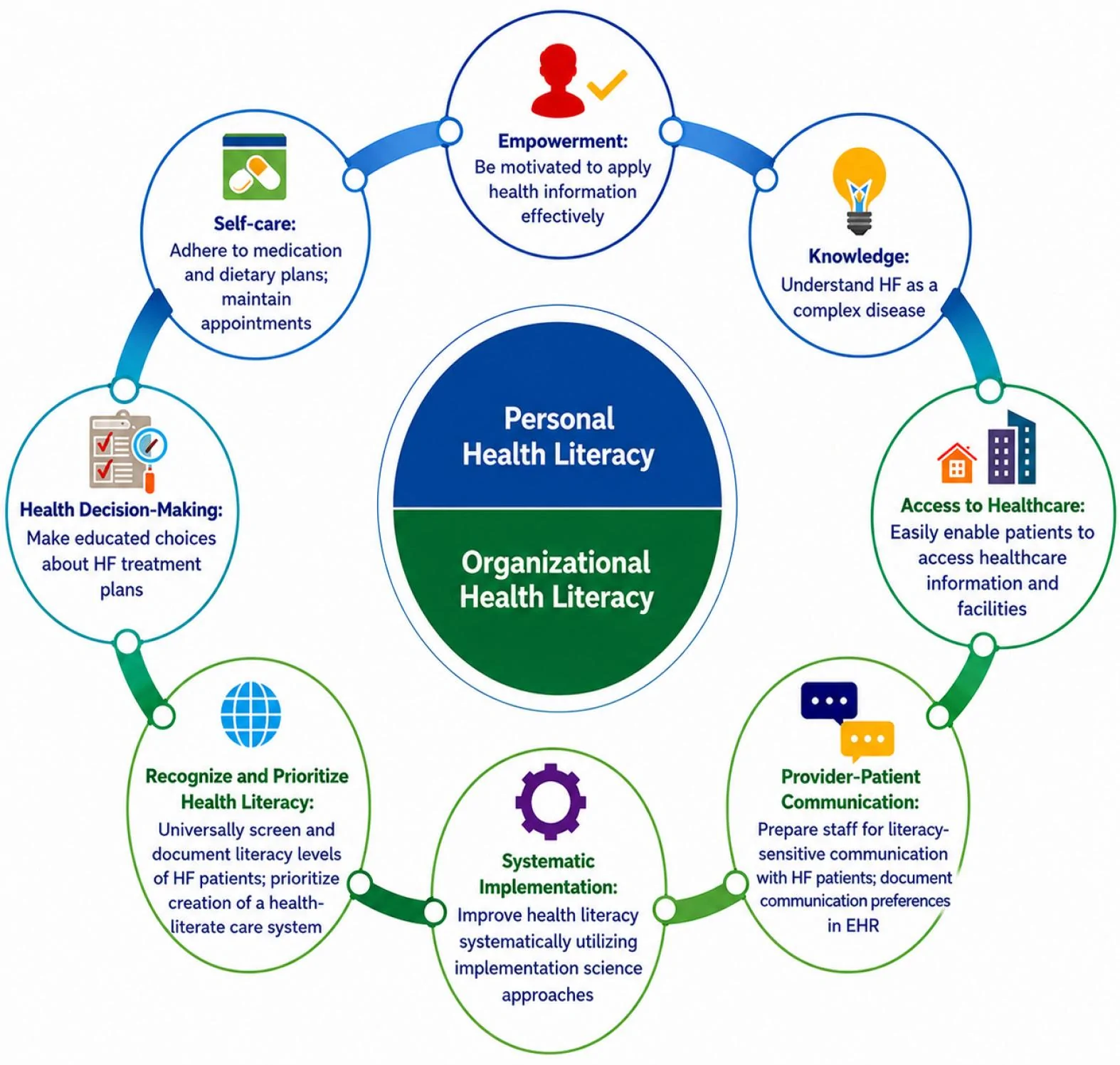
Challenges and Gaps in Health Literacy for Heart Failure Management. “Reproduced from”^2^. This figure illustrates the primary challenges related to low health literacy, including frequent hospital readmissions and diminished quality of life, among patients with heart failure, underscoring the necessity for targeted policy interventions^2^.

 Research indicates that health literacy is closely associated with medication adherence and overall quality of life.^18,19^ Furthermore, low health literacy has been linked to increased hospitalization and elevated healthcare costs.^20^

 Globally, the prevalence of heart failure has risen from 1-2% in developed adult populations to 1.26-6.7% in the Asia-Pacific region, particularly among younger and more symptomatic patients. ^21^ This trend places significant strain on the healthcare systems of developing countries, such as Iran, where disparities in access to advanced care and low health literacy further complicate disease management.

 In Iran, studies have demonstrated that educational attainment correlates with health literacy among patients with heart failure and ischemic heart disease. ^8,22^ Additional research in Iran has revealed that patients with heart failure frequently exhibit low levels of health literacy. ^4,23^ The sources from which patients obtain health information can serve as predictors of health literacy, potentially highlighting the lack of a structured, patient-centered education system (Table 2).^8^

**Table 2:** Evaluation of Policy Alternatives for the Integration of Health Literacy in Heart Failure Management

**Policy Option**	**Description**	**Advantages**	**Disadvantages**	**Primary Responsible Stakeholders / Lead Entities**
Integrating health literacy assessments into primary care practices	Implementation of a program to evaluate the health literacy levels of heart failure patients in primary care centers utilizing standardized tools, such as the Ishikawa scale (2008), to identify educational needs and allocate resources effectively.	- Early identification of patients with low health literacy, contributing to a reduction in readmission rates. - Cost-effective solutions compared to hospital interventions by leveraging existing infrastructure. - Potential for national scalability with coordinated efforts from the Ministry of Health.	- Extensive training of primary care staff is necessary, which can be both time-consuming and costly. - Infrastructure limitations exist in underserved areas. - There may be potential staff resistance resulting from an increased workload.	Ministry of Health and Medical Education (MoHME) – Primary Health Care Network Provincial Health Deputy Offices Family Physicians and Primary Care Teams
Establishing specialized clinics for health literacy education	Establish comprehensive clinics within hospitals and university centers in collaboration with the Ministry of Health and the Iranian Heart Association to offer assessment services, patient-centered education, and post-discharge follow-up care.	- Increasing adherence to treatment and enhancing quality of life through specialized training. - Strengthening coordination between primary care and hospital services. - Developing replicable models for the management of other chronic diseases.	- High costs associated with the establishment and maintenance of clinics. - Challenges in the short-term recruitment and training of specialized personnel. - Limited patient access in remote areas due to the concentration of services in urban centers.	Ministry of Health and Medical Education (MoHME) – Deputy for Treatment and Specialized Care Iranian Heart Association University of Medical Sciences / Cardiology Departments (e.g., Teaching Hospitals)
Incorporating health literacy into electronic health records (EHR)	Integration of health literacy assessment and monitoring tools into electronic health record (EHR) systems for continuous monitoring and data-driven policymaking.	- The capacity for continuous monitoring and the provision of targeted interventions based on data analysis. - Enhanced clinical decision-making through access to health literacy information. - Alignment with global trends in the digitalization of healthcare systems.	- High costs associated with the development and integration of EHR systems. - Requirement for comprehensive staff training to effectively utilize EHR systems. - Challenges related to data privacy and security.	Ministry of Health and Medical Education (MoHME) - Deputy for Management and Information Technology / National Electronic Health Record Project Office - Health Information Technology Companies / Vendors - Medical Universities (for Pilot Testing)
Developing culturally relevant and needs-based educational programs	Designing educational interventions that are customized to align with the literacy levels, ages, and cultural backgrounds of patients, utilizing straightforward tools such as videos and visual guides.	- Increased acceptance of education among patients with low literacy or diverse cultural backgrounds. - Low costs of producing digital educational content. - Widespread accessibility through digital platforms.	- Extensive research is required to adapt content to cultural differences. - Access to digital tools is limited in underserved areas. - There is a risk of producing substandard content without adequate oversight.	Ministry of Health and Medical Education (MoHME) - Health Education and Promotion Office - Iranian Heart Association - Local NGOs and Community-Based Organizations - Medical Universities - Health Promotion Researchers

 Health literacy is a critical component in the management of heart failure, significantly contributing to improved self-management, decreased hospital readmissions, and enhanced quality of life for patients.^11^ Research indicates that integrating of health literacy into healthcare systems can mitigate health inequalities and facilitate better clinical outcomes.^24^ Consequently, this policy brief aims to highlight health literacy as a key policy priority for enhancing heart failure care. This document addresses these gaps by proposing actionable strategies for incorporating health literacy into the healthcare system, thereby improving patient self-management, promoting health equity, and reducing health disparities.

## The Study

 This policy brief is based on a study involving 385 patients with heart failure who sought care at the specialized cardiology clinic of Imam Ali Hospital in Kermanshah. Participants were selected using convenience sampling.^25^ A convenience sampling method was employed because of the practical constraints inherent in recruiting a clinically well-characterized sample of patients with heart failure from a single specialized cardiology referral center. This approach is commonly utilized in hospital-based cross-sectional studies of health literacy and self-care behaviors in chronic cardiac populations, given the logistical barriers, patient vulnerability, and resource limitations. ^4,13^ However, convenience sampling may introduce selection bias and limit the generalizability of the findings to the broader Iranian population with heart failure, particularly those in rural areas or with more restricted access to specialized care. Therefore, readers should interpret the results with appropriate caution regarding external validity.

 This questionnaire was developed by Ishikawa in 2008^26^ to measure health literacy and comprises 12 items that assess three dimensions of health literacy in patients with heart failure: functional (items 1–4), communicative (items 5–8), and critical (items 9–12). Responses were scored on a 4-point Likert scale: 1 = not relevant, 2 = somewhat relevant, 3 = quite relevant, and 4 = very relevant. Each item is scored from 1 to 4, yielding a possible total scale score of 12-48. Higher scores indicate better health literacy.

 The mean health literacy (HL) score of the patients with heart failure was 38.74 ± 5.9. The mean scores across the three domains were as follows: functional health literacy, 13.54 ± 4.8; communicative health literacy, 13.63 ± 3.1; and critical health literacy, 11.55 ± 2.6. Among them, critical health literacy exhibited the lowest mean score. In Iran, Barati et al.^27^ reported composite reliability and Cronbach’s alpha coefficients for all constructs above the recommended threshold of 0.70, confirming the scale’s adequate internal consistency. The test–retest reliability ranged from 0.78 to 0.90, indicating a good level of stability over time.

 Data analysis in this descriptive cross-sectional study was conducted using descriptive statistics, including frequencies, percentages, means, and standard deviations, to summarize the sociodemographic characteristics of the sample and the levels of health literacy (total FCCHL scale score and mean scores for the functional, communicative, and critical subscales). The normality of the distribution of continuous variables was assessed using the Kolmogorov–Smirnov test. All analyses were performed using SPSS version 24, with the statistical significance level set at *P* < 0.05. Given the descriptive nature of the study and the primary focus of this policy brief on documenting health literacy levels and their policy implications for heart failure care, no advanced inferential statistical tests (such as correlation or regression analyses) were reported in the results.

 The inclusion criteria were as follows:

1. Possession of a high school diploma or higher education.
2. Age between 18 and 80 years old.
3. Heart failure was diagnosed based on clinical symptoms.
4. Electrocardiography (ECG) and echocardiographic results indicating an ejection fraction below 40%.
5. Classification as functional class II to IV was confirmed by the patient’s medical records and cardiologist.

Participants were deemed eligible if they maintained stable general conditions, demonstrated effective communication skills, and provided informed consent. The exclusion criteria were as follows unwillingness to continue participation, presence of physical or cognitive impairments, incomplete questionnaire responses, deterioration in health status, or dissatisfaction with participation. Table 1 presents the proposed policy options, along with their descriptions, advantages, and disadvantages, to facilitate informed decision-making by the policymakers.

 The data presented in Figure 3 indicate that the average health literacy level among Iranian patients with heart failure is currently suboptimal, particularly in the critical dimensions of health literacy. Implementing targeted educational programs and incorporating health literacy interventions into routine care have been effective in improving patient self-management, increasing treatment adherence, reducing hospital readmissions, and ultimately enhancing quality of life and cost-effectiveness in the management of heart failure. The notably lower score in the critical health literacy dimension (mean 11.55 ± 2.6) suggests that patients experience greater difficulty in critically evaluating health information, appraising sources, and making informed decisions regarding complex treatment options. This finding highlights the need for higher-order educational strategies that go beyond basic information provision to build skills in the critical appraisal of health information, thereby supporting more effective self-management and reducing preventable hospital readmissions.

**Figure 3:**
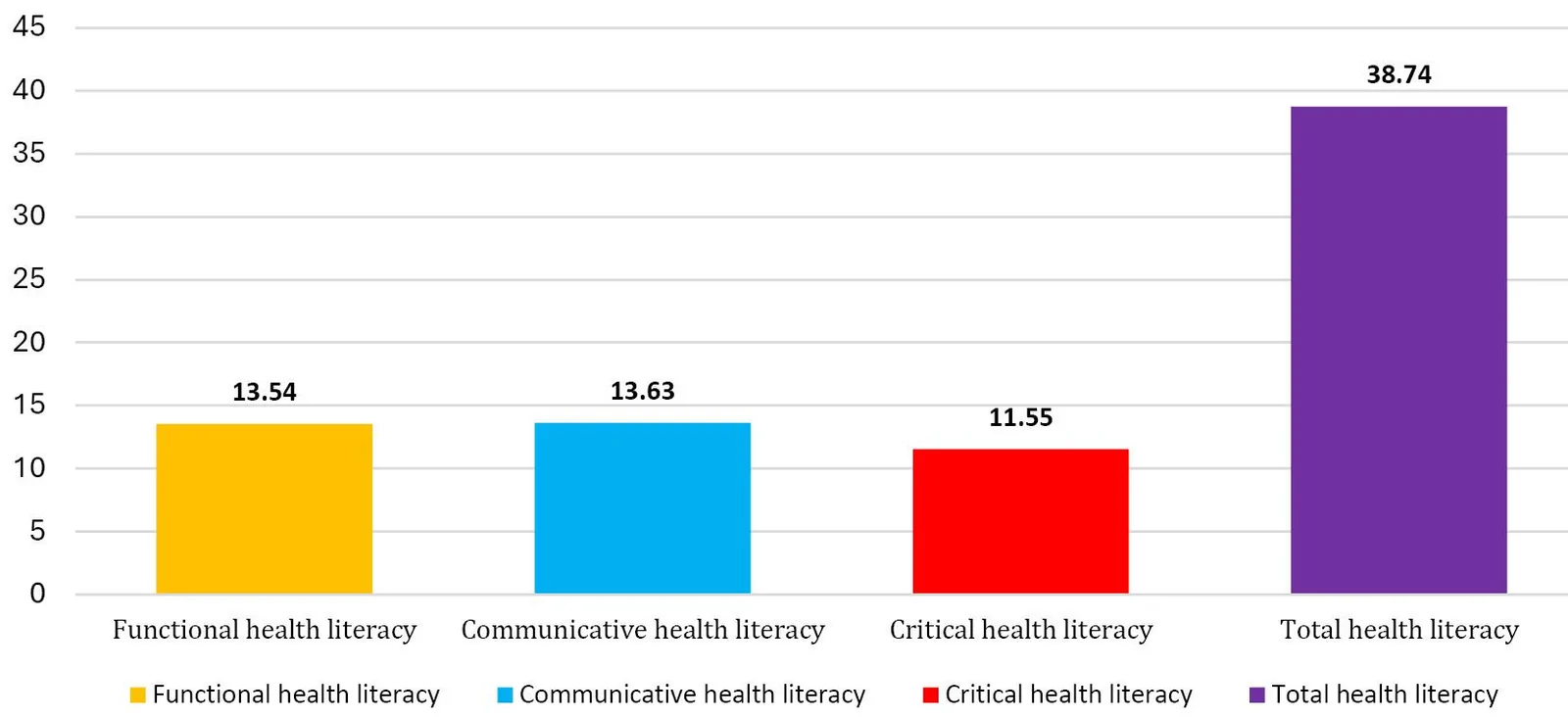
Mean Variables of Health Literacy in Patients with Heart Failure

## Limitations of the Study

 Although this study identified substantial gaps in health literacy, particularly in the critical domain, among Iranian patients with heart failure, the cross-sectional design and absence of multivariable adjustment limit our ability to isolate the independent contribution of health literacy to clinical outcomes. Several key confounding variables likely influence both health literacy levels and heart failure-related outcomes (e.g., hospital readmissions, medication adherence, and quality of life), including socioeconomic status, educational attainment, social support, multimorbidity, depression, and access to care. These factors are well documented in the literature as bidirectional determinants of health literacy and self-management capacity. ^3-4^ For instance, a lower socioeconomic status and limited social support may simultaneously reduce health literacy and increase the risk of readmission, potentially overstating the direct effect of health literacy in unadjusted analyses. Future longitudinal or multivariate studies are required to disentangle these complex relationships and quantify the unique contribution of health literacy while controlling for confounders.

## Policy Recommendations

 Key policy options and messages, which are prioritized based on the implementation timeline in the Iranian healthcare context.

## Short-term priorities (6–18 months)

 We should focus on low-cost, high-impact interventions that leverage existing infrastructure and deliver immediate benefits to patients with heart failure.

###  Integrating health literacy assessment into primary care 

 Design and implement a standardized program to routinely assess health literacy levels among all patients with HF attending primary care centers. This will enable the early identification of educational needs and facilitate targeted resource allocation.

###  Training healthcare professionals in effective communication 

 Mandatory continuing education courses for physicians and nurses should be introduced, emphasizing evidence-based communication strategies tailored to patients with low health literacy. The goal is to improve the clarity and effectiveness of health messaging and to enhance clinical outcomes.

###  Development of culturally relevant and needs-based education 

 Develop and disseminate tailored educational materials adapted to patients’ literacy levels, age groups, and cultural/ethnic backgrounds, incorporating accessible formats such as short educational videos, pictorial guides, and simple-language brochures to increase comprehension and engagement among individuals with low literacy.

###  Promoting health literacy-based cardiac rehabilitation

 Integrating structured health literacy education components into existing cardiac rehabilitation programs within heart failure management clinics can improve patients’ recognition of warning signs, symptom interpretation, and timely decision-making.

## Medium-term priorities (1.5–3 years)

 It is important to focus on building specialized capacity, expanding equitable access, and embedding health literacy into routine clinical workflows.

###  Establishing specialized health literacy education clinics

 Establish dedicated health literacy education clinics within major hospitals, drawing on successful models, such as those implemented by the Heart and Stroke Foundation of Canada. These clinics should collaborate with the Ministry of Health and Medical Education and the Iranian Heart Association to deliver individualized assessments, person-centered education, and structured post-discharge follow-ups.

###  Expanding access to digital health resources 

 Policies should be formulated to ensure equitable access to reliable, user-friendly digital health tools (mobile applications, teleconsultation platforms, and online educational content), particularly in underserved and rural regions of Iran, drawing on best practices from European initiatives to reduce geographic and socioeconomic disparities in health information access.

###  Integrating health literacy into the Electronic Health Record (EHR)

 Incorporating standardized health literacy assessment and longitudinal monitoring tools into the national Electronic Health Record system will enable continuous tracking, personalized care planning, and data-informed policymaking in heart failure management.

## Long-term priorities (3+ years)

 It focuses on systemic institutionalization, financial sustainability, and national-level evaluation mechanisms.

###  Coordination with international programs 

 Develop national clinical guidelines and standards for health literacy education in cardiac care, aligned with the World Health Organization’s Global Action Plan for the Prevention and Control of Non-communicable Diseases and the Amsterdam Declaration (International Health Literacy Association & WHO).

###  Financial and insurance support for health literacy interventions 

 Advocate for the inclusion of health literacy education services and related rehabilitation interventions in public and private health insurance benefit packages to reduce financial barriers and promote equitable access for vulnerable populations.

###  Monitoring and Evaluation of National Policies 

 Establish a comprehensive, multi-stakeholder, national monitoring and evaluation framework for health literacy policies. This system should integrate key performance indicators (e.g., readmission rates, medication adherence scores, and patient-reported health literacy levels) into routine health system performance assessments to ensure long-term effectiveness, accountability, and continuous quality improvement.

 These prioritized recommendations reflect a staged, pragmatic approach that begins with feasible, immediate actions and progressively builds toward the sustainable, system-wide integration of health literacy as a core component of heart failure care in Iran.

## Financial Feasibility and Cost-Benefit Considerations for National Rollout

 The proposed policy recommendations, particularly short- and medium-term actions such as integrating health literacy screening into primary care, training healthcare professionals, and developing low-cost, culturally adapted educational materials, are financially feasible within the Iranian context, as they primarily leverage existing infrastructure, including the primary healthcare network, cardiac rehabilitation programs, and the current workforce. International evidence suggests that initial investments in health literacy interventions for patients with heart failure are generally offset within 12 to 18 months through significant reductions in preventable readmissions, emergency service utilization, and costs associated with medication non-adherence (Heart and Stroke Foundation of Canada model indicated a 22% reduction in readmissions). ^3^ For national scale-up in Iran, key cost drivers would include staff training (short-term), the production of multilingual and digital educational content (medium-term), and electronic health record integration (long-term). However, the return on investment (ROI) is anticipated to be favorable, with projected reductions of 15 to 30% in avoidable hospitalizations, a significant contributor to the economic burden of heart failure. Strategic budget reallocation from existing non-communicable disease (NCD) prevention and health promotion funds, combined with advocacy for insurance reimbursement of health literacy services, would further enhance the financial sustainability and feasibility of this initiative.

## Concrete Examples and Pilot Initiatives

 To illustrate the feasibility and contextual relevance, the following real-world examples can inform implementation:

**Short-term priorities:** A home-based educational intervention involving community health volunteers (CHVs) in western Iran (including rural areas of Kermanshah province) successfully improved self-care behaviors and reduced the risk of readmission in patients with chronic heart failure through simple, localized training delivered by trained volunteers (randomized trial in western Iran). Similarly, culturally adapted pictorial and video-based materials tested in multi-ethnic rural settings (e.g., Kurdish and Azeri regions) have been found to be more acceptable among low-literacy patients^28^.

**Medium-term priorities:** Pilot implementation of specialized post-discharge education clinics, modeled on the Heart and Stroke Foundation of Canada^29^ (with documented 22% readmission reduction), could begin in major Iranian university hospitals (e.g., Imam Ali Hospital in Kermanshah or Tehran-based centers). Partnerships with local NGOs for distributing mobile/digital health content in underserved rural provinces (drawing from successful virtual education pilots in Iranian heart failure cohorts) could address geographic barriers.

**Long-term priorities:** National guideline development could build on existing community-based NCD education initiatives in Iran, while insurance advocacy might reference successful NGO-led rural health promotion programs that secured partial reimbursement for patient education services.

## Alignment with Iran’s National Development Plans and Health System Reforms

 The policy recommendations outlined in this brief are fully aligned with Iran’s overarching national health policy. The Health Transformation Plan (HTP), launched in 2014, prioritizes universal health coverage (UHC), reduction of out-of-pocket payments, strengthening primary health care (PHC), and better management of non-communicable diseases (NCDs), including heart failure, as a major contributor to the NCD burden. Integrating health literacy assessment and promotion directly supports the HTP’s goals of equity, quality improvement, and patient empowerment. Furthermore, the Seventh National Development Plan (2023–2027), particularly in its chapter on health system improvement (e.g., Article 68 and related provisions), emphasizes prevention, community education, chronic disease management, and financial sustainability, explicitly recognizing health literacy as a critical enabler for reducing disease burden and enhancing system efficiency. Short-term actions (e.g., primary care screening and professional training) can serve as immediate extensions of HTP’s PHC focus, while medium- and long-term recommendations (specialized clinics, insurance coverage, and national monitoring) contribute to the Plan’s objectives of sustainable, equitable, and data-driven health system advancement.

## Iran’s Specific Considerations for Integrating Health Literacy in Heart Failure Management

 Iran, characterized by its extensive cultural and geographical diversity, encounters distinct challenges in integrating health literacy within the healthcare system for heart failure patients.^4^ This integration demands focused policy-making and careful consideration of local contexts. The implementation of health literacy programs in underserved and rural regions of Iran is hindered by multiple challenges at the individual, interpersonal, organizational, and societal levels, necessitating targeted policy solutions.^30^ Furthermore, Iran’s cultural and linguistic diversity, which includes various ethnic groups such as Kurds, Lurs, Turks, and Baloch, along with the differing literacy levels between urban and rural populations, presents significant challenges in designing uniform educational interventions. Patients in regions with low literacy or diverse cultural backgrounds may not respond effectively to educational content that is not communicated in a straightforward language and adapted to the local culture. For instance, a study conducted in Iran indicated that individuals from different cultural backgrounds possess varying interpretations of health concepts, which influences their acceptance of medical education.^4^

 To address these challenges, the following recommendations are proposed.

- **Enhance Local Infrastructure:** Invest in the development of digital infrastructure and provide training for personnel in underserved areas to implement health literacy programs, focusing on primary care centers.
- **Develop Culturally Centered Interventions:** Develop multilingual educational materials that are culturally relevant, incorporating Indigenous stories or proverbs to facilitate acceptance among diverse ethnic groups.
- **Facilitate Community Engagement:** Collaborate with local leaders and non-governmental organizations to promote health literacy through community-based initiatives, particularly in rural areas where access to health services is constrained.

## Conclusion

 Health literacy is a critical policy imperative in heart failure management. The systematic integration of health literacy into cardiac care frameworks empowers patients, strengthens self-management behaviors, and improves treatment adherence, thereby effectively reducing preventable hospital readmissions, lowering healthcare costs, and advancing health equity. The evidence synthesized in this policy brief, drawn from both national and international studies, demonstrates that targeted, needs-based educational programs, enhanced communication training for healthcare professionals, and equitable access to reliable digital health resources can yield substantial clinical and economic benefits for individuals living with heart failure. In the Iranian context, the establishment of specialized health literacy education clinics for cardiac patients (modeled on successful international frameworks such as the WHO Global Action Plan for the Prevention and Control of Noncommunicable Diseases) and the incorporation of routine health literacy assessment and monitoring into electronic health record systems would constitute a strategic step toward achieving more sustainable and equitable healthcare delivery. Strengthening health literacy must be institutionalized as a cross-sectoral policy priority. Beyond its technical and clinical value, this represents an ethical obligation for health-system planning. Improved population health literacy has the potential to reduce the overall burden of disease, enhance healthcare system efficiency, and contribute meaningfully to health-related Sustainable Development Goals. A key limitation of this study is its cross-sectional design, which precludes any conclusions regarding causality. While the observed low levels of health literacy — particularly in the critical domain — are strongly associated with known challenges in heart failure management, the directionality and mechanistic pathways remain to be elucidated in future longitudinal research. Therefore, the policy recommendations presented here are grounded in robust associational evidence and international best practices but should be implemented and evaluated with an awareness of this inherent limitation. However, additional longitudinal research is necessary to rigorously assess the long-term effects of health literacy-based interventions on key clinical outcomes in patients with heart failure within the Iranian healthcare context. However, further longitudinal research is essential to rigorously evaluate the long-term effects of health literacy-based interventions on key clinical outcomes in patients with heart failure within the Iranian healthcare context.

## Competing Interests

 None to declare.

## Ethical Approval

 Ethical approval was obtained and approved for the study from the Ethics Committee at the Kermanshah University of Medical Sciences. The ethics code for this study was IR.KUMS.REC.1404.408. Informed consent was obtained from the participants, their parents, and legally authorized representatives. This Policy Brief is based on the findings of research project No. 4040633, approved by the Ethics Committee of Kermanshah University of Medical Sciences, Kermanshah, Iran.

## References

[1] Shahim B, Kapelios CJ, Savarese G, Lund LH (2023). Global public health burden of heart failure: an updated review. Card Fail Rev.

[2] Ionescu R, Allen LA, Breathett K, Fowler BK, Jackson EA, Kundrick J (2025). Health literacy in heart failure: a review of the gaps and challenges. JACC Adv.

[3] Fabbri M, Murad MH, Wennberg AM, Turcano P, Erwin PJ, Alahdab F (2020). Health literacy and outcomes among patients with heart failure: a systematic review and meta-analysis. JACC Heart Fail.

[4] Rezaei S, Vaezi F, Afzal G, Naderi N, Mehralian G (2022). Medication adherence and health literacy in patients with heart failure: a cross-sectional survey in Iran. Health Lit Res Pract.

[5] Tan SC, Zheng BB, Tang ML, Chu H, Zhao YT, Weng C (2025). Global burden of cardiovascular diseases and its risk factors, 1990-2021: a systematic analysis for the Global Burden of Disease Study 2021. QJM.

[6] Heidari-Foroozan M, Farshbafnadi M, Golestani A, Younesian S, Jafary H, Rashidi MM (2025). National and Subnational Burden of Cardiovascular Diseases in Iran from 1990 to 2021: results from Global Burden of Diseases 2021 Study. Glob Heart.

[7] Torabi Z, Shakibazadeh E, Tajvar M, Rezaei N (2025). Non-communicable diseases challenges and opportunities in Iran: a qualitative study. Sci Rep.

[8] Adib-Hajbaghery M, Hakimzadeh Z, Adib ME (2018). Health literacy in patients with ischemic heart diseases. J Health Lit.

[9] World Health Organization (WHO). Health Literacy in the Context of Health, Well-Being and Learning Outcomes the Case of Children and Adolescents in Schools: The Case of Children and Adolescents in Schools. WHO; 2021. p. 1-27. Available from: https://iris.who.int/server/api/core/bitstreams/31888cbe-60b5-499d-8953-ee61d7535b6b/content.

[10] Nakon O, Utriyaprasit K, Wanitkun N, Sindhu S, Viwatwongkasem C, Tankumpuan T (2024). The effect of health literacy on health status in patients with heart failure: a path analysis. J Multidiscip Healthc.

[11] Van Driel AG, Heide J, Klis-Busink AM, Van Staa AL, Van Hooft SM, Jaarsma T. Health literacy and self-care in patients with heart failure: a cross-sectional study. Eur J Cardiovasc Nurs 2025;24(Suppl 1):zvaf122.084. doi: 10.1093/eurjcn/zvaf122.084.

[12] Pedro AR, Raposo B, Brandão D, Martinho H, Almeida M, Pardal M, et al. Health literacy and individuals’ perception regarding heart failure management: patient perspective. Eur J Public Health 2024;34(Suppl 3):ckae144.1702. doi: 10.1093/eurpub/ckae144.1702.

[13] Son YJ, Shim DK, Seo EK, Seo EJ (2018). Health literacy but not frailty predict self-care behaviors in patients with heart failure. Int J Environ Res Public Health.

[14] Cajita MI, Cajita TR, Han HR (2016). Health literacy and heart failure: a systematic review. J Cardiovasc Nurs.

[15] Le C, Straßmayr C, Giraudeau N, Cresswell-Smith J, Barailla C, Berrefjord PR (2025). Strengthening population and organizational health literacy to reduce social inequalities within the JA PreventNCD. Scand J Public Health.

[16] Jovanić M, Zdravković M, Stanisavljević D, Jović Vraneš A (2018). Exploring the importance of health literacy for the quality of life in patients with heart failure. Int J Environ Res Public Health.

[17] Osborne RH, Elmer S, Hawkins M, Cheng CC, Batterham RW, Dias S (2022). Health literacy development is central to the prevention and control of non-communicable diseases. BMJ Glob Health.

[18] Bektemur G, Cortuk M, Keles E, Baydili KN (2025). Association of health literacy and medication adherence with quality of life in COPD patients. P R Health Sci J.

[19] Vater M, Davis A, Jaser S (2024). Evaluation of health literacy and its association with medication adherence and quality of life in childhood-onset systemic lupus erythematosus. Lupus.

[20] Koerts ND, Horváth B (2025). Exploring health literacy among adults with hidradenitis suppurativa. Dermatology.

[21] Rajadurai J, Tse HF, Wang CH, Yang NI, Zhou J, Sim D (2017). Understanding the epidemiology of heart failure to improve management practices: an Asia-Pacific perspective. J Card Fail.

[22] Sheikh Sharafi H, Seyed Amini B (2017). Assessment of health literacy and self-care in heart failure patients. J Health Lit.

[23] Marzangi A, Ahangarzadeh Rezaei S, Ghareagaji Asl R (2018). Health literacy in heart disease community that covered by health services centers in south of West Azerbaijan province. Nurs Midwifery J.

[24] Jolidon V, Eicher M, Peytremann-Bridevaux I, Arditi C (2024). Inequalities in patients’ experiences with cancer care: the role of economic and health literacy determinants. BMC Health Serv Res.

[25] Darabi F, Montazeri N, Bakhshi S, Ziapour A, Ghasemi Tahereh. Investigating the relationship between health literacy and perceived social support in patients with heart failure: a cross-sectional study in Northwest Iran. Unpublished manuscript under peer review. 2026.

[26] Ishikawa H, Takeuchi T, Yano E (2008). Measuring functional, communicative, and critical health literacy among diabetic patients. Diabetes Care.

[27] Barati M, Taheri-Kharameh Z, Farghadani Z, Rásky É (2019). Validity and reliability evaluation of the Persian version of the heart failure-specific health literacy scale. Int J Community Based Nurs Midwifery.

[28] Siabani S, Driscoll T, Davidson PM, Leeder SR (2016). Efficacy of a home-based educational strategy involving community health volunteers in improving self-care in patients with chronic heart failure in western Iran: a randomized controlled trial. Eur J Cardiovasc Nurs.

[29] Jalloh MB, Averbuch T, Kulkarni P, Granger CB, Januzzi JL, Zannad F (2023). Bridging treatment implementation gaps in patients with heart failure: JACC focus seminar 2/3. J Am Coll Cardiol.

[30] Darabi F, Ziapour A, Mohammadkhah F, Yıldırım M, Nejhaddadgar N (2025). Barriers to the development of health literacy in Iran’s health system: a qualitative study. Health Lit Res Pract.

